# Impact of ABO incompatible kidney transplantation on living donor transplantation

**DOI:** 10.1371/journal.pone.0173878

**Published:** 2017-03-21

**Authors:** Ji Hyun Yu, Byung Ha Chung, Chul Woo Yang, for the Korean Organ Transplantation Registry Study Group

**Affiliations:** Transplantation research center, Division of Nephrology, Department of Internal Medicine, Seoul St. Mary’s Hospital, College of Medicine, The Catholic University of Korea, Seoul, Korea; University of Edinburgh MRC Centre for Inflammation Research, UNITED KINGDOM

## Abstract

**Background:**

ABO incompatible kidney transplantation (ABOi-KT) is an important approach for overcoming donor shortages. We evaluated the effect of ABOi-KT on living donor KT.

**Methods:**

Two nationwide transplantation databases were used. We evaluated the impact of ABOi-KT on overall living donor transplant activity and spousal donation as subgroup analysis. In addition, we compared the clinical outcome between ABOi-KT and ABO compatible KT (ABOc-KT) from spousal donor, and performed a Cox proportional hazards regression analysis to define the risk factors affecting the allograft outcomes.

**Result:**

The introduction of ABOi-KT increased overall living donor KT by 12.2% and its portion was increased from 0.3% to 21.7% during study period. The ABOi-KT in living unrelated KT was two times higher than that of living related donor KT (17.8 vs.9.8%). Spousal donor was a major portion of living unrelated KT (77.6%) and ABOi-KT increased spousal donation from 10% to 31.5% in living donor KT. In addition, increasing rate ABOi-KT from spousal donor was 10 times higher than that of living related donor. The clinical outcome (incidence of acute rejection, allograft function, and allograft and patient survival rates) of ABOi-KT from spousal donor was comparable to that of ABOc-KT. Neither ABO incompatibility nor spousal donor was associated with acute rejection or allograft failure on multivariate analysis.

**Conclusions:**

ABOi-KT increased overall living donor KT, and ABOi-KT from spousal donor is rapidly increasing with favorable clinical outcomes.

## Introduction

During the past three decades, the structure of the Korean family has changed from large families to nuclear families. Additionally, the birth rate per one reproducible woman has decreased significantly from 2.82 in 1980 to 1.2 in 2014.[1] This has led to the gradual decrease in the number of potential living related donors including sibling or offspring donors, and spouses have become important alternatives to living related donors for kidney donation in Korea.[2, 3]

ABO blood type incompatibility was an important barrier of kidney transplantation (KT). We previously reported that the most common reason for the enrollment of donor kidney exchange program was ABO blood type incompatibility and the most common intended donor-recipient relationship was spousal.[4] Therefore, it is expected that the introduction of ABO incompatible KT (ABOi-KT) will enable those patients to undergo KT, which will contribute to overcome donor kidney shortage.

In Korea, the ABOi-KT started in 2007 and has rapidly increased since then.[5] Thus, the influence of ABOi-KT on overall KT activity, especially spousal donor KT has been interest. In addition, we interested in clinical outcomes of ABOi-KT from spousal donors as compared with the ABO compatible KT (ABOc-KT) from spousal donors. To evaluate these parameters, we used nationwide transplantation database. This study was aimed to provide a rationale for KT in end-stage renal disease patients whose only potential donors are ABO incompatible spouses.

## Materials and methods

### Study population

We used two databases in this study. The first database from Korean Network for Organ Sharing was used to evaluate the effect of ABOi-KT on number of KT. It contains all the KT cases in Korea since 2000 (S1 File).^5^ Among these cases, we used data from 2003 to 2014 (the period for which donor information was fully satisfied). The second database was the Korean Organ Transplantation Registry established by the Korean Society for Transplantation [6, 7], to evaluate clinical outcomes of ABOi-KT from spousal donors. A total of 4,987 cases from 46 KT centers between 2009 and 2012 were included, which comprised 92.1% of the KTs in Korea during this period. Annual report of Korean Network for Organ Sharing is available at http://www.konos.go.kr/konosis/common/bizlogic.jsp (accessed 10th), and Korean Organ Transplantation Registry of the Korean Society for Transplantation is available at http://www.kotry.org.

We divided the patients into ABOi-KT from spousal donors, ABOc-KT from spousal donors and living related donor KT. The baseline characteristics of each group are presented in Table 1. The age or mismatched number of human leukocyte antigen (HLA) of recipients and donors of ABOi-KT and ABOc-KT from spousal donors were older or higher than the living related donor KT. There was no difference in the proportion of re-transplantation, donor specific antibody positivity and cross-match positivity. The tacrolimus-based maintenance immunosuppression including mycophenolic acid and steroids was higher in the ABOi-KT than the ABOc-KT and living related donor. This study was approved by the Institutional Review Board (IRB) of the Seoul St. Mary’s Hospital (KC12RCMI0203) and has been conducted according to the principles expressed in the Declaration of Helsinki.

**Table 1:** Baseline patient characteristics.

	ABOi-SD-KT (n = 150)	ABOc-SD-KT (n = 566)	LRD-KT(n = 2112)	*p*-value
Recipient age, years, mean ± s.d.	48.0 ± 8.2 ^*a*^	48.4 ± 8.0 ^*a*^	41.0 ± 13.3	< 0.001
Donor age, years, mean ± s.d.	47.0 ± 8.3 ^*a*^	47.0 ± 8.0 ^*a*^	39.7 ± 11.9	< 0.001
Recipient, male, n (%)	106 (70.7)	380 (67.1)	1194 (56.5)	< 0.001
Donor, male, n (%)	44 (29.3)	186 (32.9)	1082 (51.2)	< 0.001
Retransplantation, n (%)	12 (8.0)	27 (4.8)	107 (5.1)	0.209
Duration of dialysis, months, median (IQR)	8.5 (32)	3.0 (21)	4.0 (21)	0.035
Causes of ESRD				< 0.001
Chronic glomerulonephritis, n (%)	44 (29.3)	136 (24.0)	696 (33.0)	
Diabetes mellitus, n (%)	32 (21.3)	150 (26.5)	334 (15.8)	
Hypertension, n (%)	13 (8.7)	64 (11.3)	205 (9.7)	
Polycystic kidney disease, n (%)	15 (10.0)	44 (7.8)	51 (2.4)	
Others, n (%)	6 (4.0)	31 (5.5)	215 (10.2)	
Unknown, n (%)	40 (26.7)	141 (24.9)	611 (28.9)	
HLA mismatched number, mean ± s.d.	4.9 ± 0.9 ^*a*^	4.5 ± 1.2 ^*a*^	2.4 ± 1.4	< 0.001
Panel reactive antibody > 50%, n (%)	6 (4.8)	41 (8.7)	200 (11.5)	0.023
Presence of donor specific antibody, n (%)	11 (8.9)	30 (7.1)	116 (7.1)	0.75
Positive T cell crossmatch, n (%)	10 (6.7)	16 (2.8)	83 (3.9)	0.09
Positive B cell crossmatch, n (%)	6 (4.4)	7 (1.4)	43 (2.3)	0.105
Induction immunosuppression				0.003
Basiliximab, n (%)	134 (89.3)	502 (88.8)	1759 (83.3)	
Anti-thymocyte globulin, n (%)	9 (6.0)	17 (3.0)	72 (3.4)	
Other, n (%)	0	0	7 (0.3)	
No induction immunosuppression, n (%)	7 (4.7)	46 (8.1)	274 (13.0)	
Maintenance immunosuppressive regimen				0.001
TAC + MMF + steroid, n (%)	136 (91.3)	391 (69.4)	1415 (67.2)	
TAC + other antimetabolite + steroid, n (%)	4 (2.7)	23 (4.1)	78 (3.7)	
TAC + steroid, n (%)	0	15 (2.7)	82 (3.9)	
CsA + MMF + steroid, n (%)	7 (4.7)	90 (16.0)	400 (19.0)	
TAC + MMF, n (%)	0	6 (1.1)	19 (0.9)	
CsA + mTORi + steroid, n (%)	0	6 (1.1)	16 (0.8)	
TAC + mTORi + steroid, n (%)	0	10 (1.8)	13 (0.6)	
Other, n (%)	2 (1.3)	22 (3.8)	83 (4.9)	
Duration of follow-up, months, mean ± s.d.	21.8 ± 9.9 ^*a*^	25.3 ± 9.9 ^*a*^	26.5 ± 10.0	< 0.001

^*a*^: *P* < 0.05 vs. LRD-KT group

ABOi = ABO incompatible, SD = spousal donor, KT = kidney transplantation, ABOc = ABO compatible, LR = living related donor, s.d. = standard deviation, IQR = interquartile range, ESRD = end-stage renal disease, HLA = human leukocyte antigen, TAC = tacrolimus, MMF = mycophenolate mofetil, CsA = cyclosporine A, mTORi = mammalian target of rapamycin inhibitor.

### ABO Incompatible KT protocol

The preconditioning protocols were highly uniform across the centers.[8] They consisted of rituximab, plasmapheresis and intravenous immunoglobulin. Rituximab was used in all centers. Most centers used a single dose of 375 mg/m^2^ or 500 mg/body at the initiation of their program, but the dose tended to be reduced later to 200 mg/body or 100 mg/m^2^. Pre-transplant plasmapheresis was routinely performed in all patients. One plasma volume was exchanged with either albumin solution or fresh frozen plasma by the conventional method in most patients, but in a minority of patients, double filtration plasmapheresis was also used. Intravenous immunoglobulin (100 or 200 mg/kg) was administered after plasmapheresis in all but one center.

The target anti-A/B antibody titer on the transplant day was 1:8 or 1:16. Post-transplant preemptive plasmapheresis during the first two weeks was not performed routinely, but as needed, in patients with high anti-A/B titer or rising creatinine while awaiting the biopsy result. Tacrolimus-based triple immunosuppressants were the most popular regimen and started with the initiation of pre-transplant plasmapheresis. The target trough level of tacrolimus during the first post-transplant month was 8–12 ng/mL, and the dose of mycophenolate mofetil during the first month was 1.5 g/day in most patients. Interleukin-2 receptor blockade (97%) was used as an induction. Most of the centers adopted infection prophylaxis for *Pneumocystis jirovecii* and cytomegalovirus infection. The anti-A or anti-B titer was measured using the saline method for IgM and indirect Coombs’ test for IgG.

### Evaluation of clinical outcomes

The clinical outcome of ABOi-KT from spousal donors was evaluated in terms of the biopsy-proven acute rejection (BPAR)-free survival rate, the allograft and patient survival rates and renal allograft function assessed by the estimated glomerular filtration rate (eGFR), compared to those of the ABOc-KT and living related donor KT. BPAR was diagnosed according to Banff 2007 classification.[9] Serum creatinine levels were collected every 6 months post-transplantation, and the eGFR for each concordant time was assessed using the CKD-EPI equation.[10] BPAR-free survival was defined as the time from transplantation to the first episode of BPAR. Patient survival was defined as the time from transplantation until death from any cause.

Additionally, we compared the clinical outcomes of ABOi-KT (128 of 150) and ABOc- KT (256 of 566) after propensity score matching. It was performed using donor age, donor gender, recipient age, causes of ESRD, re-transplantation and sensitization (defined as a positive panel reactive antibody with positive cross-match or the presence of donor specific antibodies as covariates. A 1:2 nearest neighbor matching algorithm was used when the calculated propensity score was matched.

### Statistical analyses

Trends in the number of ABOi donor and spousal donor in temporal relation were analyzed using the Cochrane-Armitage trend test. Associations of year with the percent increase of spousal donor KT and living related donor KT were compared by multiple linear regression analysis. Continuous data were presented as the mean ± standard deviation (or standard error) or the median with the interquartile range according to their distribution. The data were compared using an ANOVA with post hoc analysis, Student’s t-test or the Mann-Whitney test, depending on the data type. Categorical data were compared using χ^2^ tests or Fisher’s exact tests. Kaplan-Meier curves and log-rank tests were used to describe and compare the BPAR-free survival, graft survival and patient survival rates. To define the risk factors affecting the allograft outcomes, a Cox proportional hazards regression analysis was used. A P-value < 0.05 was considered statistically significant. All of the statistical analyses were performed using SPSS (IBM SPSS Statistics, version 22).

## Results

### Effect of the ABOi-KT on overall living donor transplant activity

**Fig 1:**
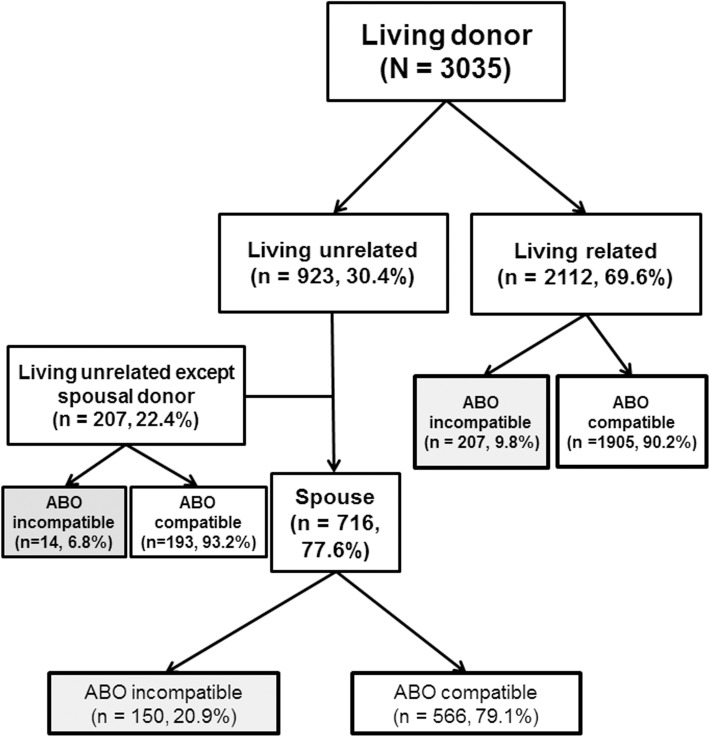
Number and proportion of ABOi-KT in living donor KT. Total 371 ABOi-KT was performed in 3045 living door KT. Living related donor KT was 55.4% (n = 207) and living unrelated donor KT was 45.6% (n = 164). Proportion of ABOi-KT in living unrelated donor KT was two times higher than living related donor KT. Spouse was a major donor source of living unrelated donor (77.6%), and its proportion of ABOi-KT was 20.9%.

Fig 1 shows the transplant activity of living donor KT and ABOi-KT during study period. Total 3035 living donor KT was performed, and living related donor KT comprised almost 70%. Total 371 ABOi-KT was performed and its proportion in living donor KT was 12.3%. The number of ABOi-KT in living unrelated donor KT was less than living related donor KT (164 vs. 207) but proportion of ABOi-KT was two times higher than living related donor KT (17.8 vs. 9.8%). Spouse was a major source of living unrelated donor (77.6%), and the proportion of ABOi-KT was 20.9%.

### Annual increase of living donor KT and spousal donation with introduction of ABOi-KT

**Fig 2:**
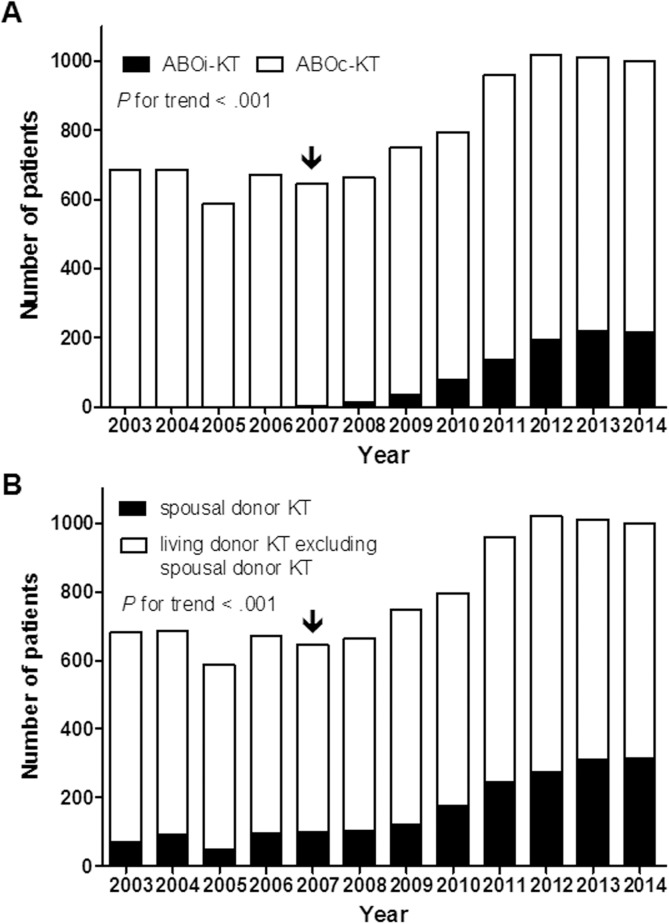
Annual increase of the ABOi-KT and spousal donor KT. (A) The annual number of ABOi-KT. (B) The annual number of spousal donor KT. Note that both living donor KT and spousal donor KT was increased annually after introduction of ABOi-KT. Arrow indicates the starting year of ABOi-KT. ABOi, ABO incompatible; KT, kidney transplantation

Fig 2 shows that annual increase of ABOi-KT and spousal donor KT. ABOi-KT was only 2 cases (0.3%) in 2007, but the number of ABOi-KT rapidly increased to 217 cases in 2014, which accounted for 21.7% of the total living donor KT (Fig 2A). Fig 2B shows annual increase of spousal donor KT with introduction of ABOi-KT. The spousal donor KT comprised about 10% of living donor KT before introduction of ABOi-KT, but its proportion increased rapidly up to 31.5% of the total living donor KT with introduction of ABOi-KT.

### Effect of ABOi-KT on spousal and living related donor KT

**Fig 3:**
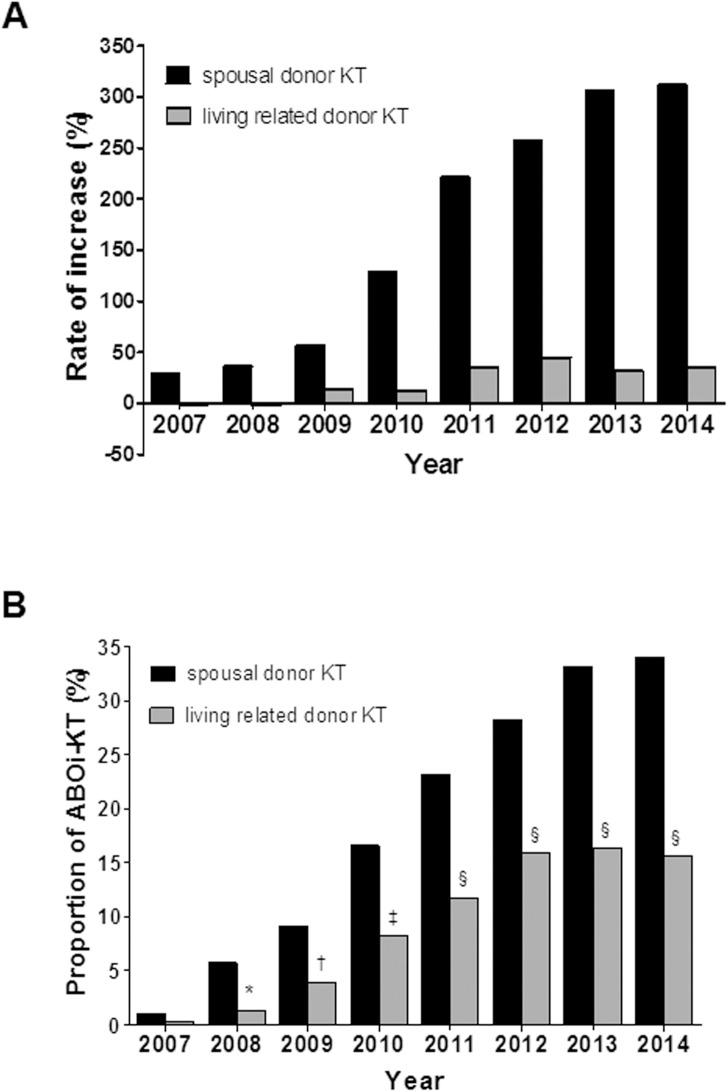
Impact of the ABOi-KT on spousal donor KT and living related donor KT. (A) Comparisons of the rate of increase between spousal donor KT and living related donor KT. Note that the percent increase of spousal donor KT was significant relative to that of living related donor KT (β ± SE: 0.02 ± 0.002, partial R^2^ 0.952, *P* < 0.001) (B) Comparison of the proportion of ABOi-KT in spousal donor KT and living related donor KT. Note that higher proportion of ABOi-KT in spousal donor KT than that of living related donor KT each year. ^*^, *P* = 0.013; ^†^, *P* = 0.017; ^‡^, *P* = 0.001; ^§^, *P* < 0.001. ABOi, ABO incompatible; KT, kidney transplantation; ABOc, ABO compatible.

Fig 3 shows the effect of ABOi-KT on transplant activity between spousal and living related donors. With introduction of ABOi-KT, both spousal donor and living related donor KT have increased. But, spousal donor KT was rapidly increased as compared with living related donor KT (312% vs. 35%, β ± SE: 0.02 ± 0.002, partial R^2^ 0.952, *P* < 0.001 for the increased rate of spousal donor KT, Fig 3A) and its proportion was also increased as compared with living related donor KT (*P* < 0.05 for each year, Fig 3B).

### BPAR episodes between ABOi-KT and ABOc-KT from spousal donor

**Fig 4:**
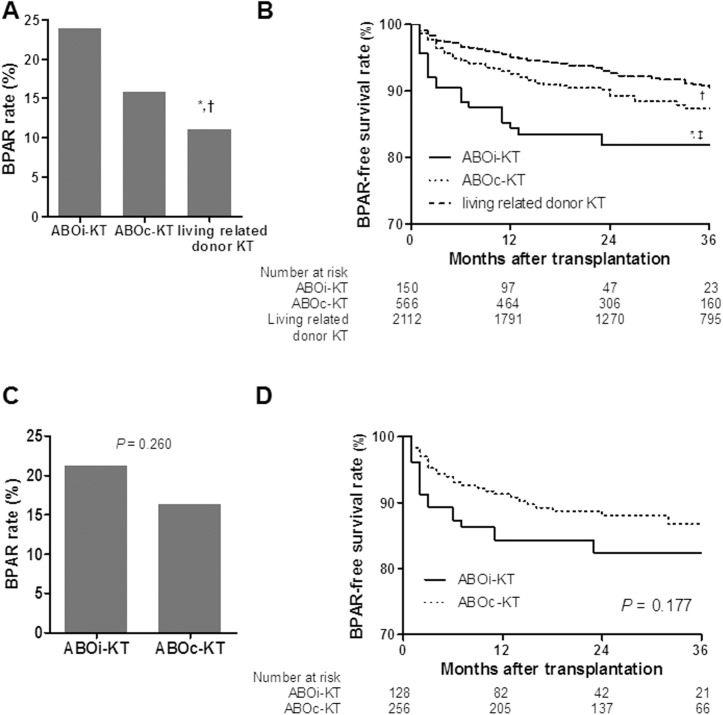
Comparisons of the BPAR between ABOi-KT and ABOc-KT from spousal donors. (A) Overall incidence of BPAR. (B) BPAR-free survival rate. (C) Incidence of BPAR after the propensity score-matching analysis. (D) BPAR-free survival rate after the propensity score-matching analysis. Note that BPAR or BPAR-free graft survival rate were not significantly different between ABOi-KT and ABOc-KT from spousal donors ^*^, *P* < 0.05 for ABOi-KT from spousal donor vs. living related donor KT; ^†^, *P* < 0.05 for ABOc-KT from spousal donor vs. living related donor KT; ^‡^, *P* < 0.05 for ABOi-KT vs. ABOc-KT from spousal donor. BPAR, biopsy-proven acute rejection; ABOi, ABO incompatible; KT, kidney transplantation; ABOc, ABO compatible.

The incidence of BPAR in the ABOi-KT and ABOc-KT from spousal donors was not significantly different (23.9 vs. 15.8%, *P* = 0.081, Fig 4A). The BPAR-free survival rates at 1, 2, and 3-year post-transplantation in the ABOi-KT (77.8, 74.4 and 74.4%, respectively) were lower than the ABOc-KT (87.6, 84.9 and 82.2%, respectively, *P* = 0.033, Fig 4B) but the comparative analysis using a propensity score-matching algorithm revealed no difference between two groups in terms of overall incidence of BPAR (21.2 vs. 16.3%, Fig 4C), and the BPAR-free survival rate (77.3 vs. 82.2% at 3-year post-transplantation, *P* = 0.177, Fig 4D). In the multivariate analysis, donor age and the degree of HLA mismatch were independent risk factors for BPAR. SD and ABO incompatibility were risk factors for BPAR in univariate analysis but not in multivariate analysis (*P* = 0.275 and *P* = 0.05 respectively, Table 2).

**Table 2:** Univariate and multivariate cox regression analysis of risk factors for biopsy-proven acute rejection.

	Univariate				Multivariate			
	HR	95% CI	95% CI	*P-*value	HR	95% CI	95% CI	*P* -value
		Lower	Upper			Lower	Upper	
Recipient age (per 1 year-old)	0.995	0.986	1.003	0.206				
Donor age (per 1 year-old)	1.017	1.007	1.026	0.001	1.017	1.002	1.032	0.022
Female recipient (vs. male)	0.831	0.666	1.037	0.102				
Female donor (vs. male)	1.125	0.908	1.393	0.281				
Retransplantation (vs. first)	0.693	0.389	1.233	0.212				
Duration of dialysis (per month)	1	0.997	1.004	0.812				
Causes of ESRD								
Diabetes mellitus	Referent							
Hypertension	0.685	0.449	1.047	0.08	0.468	0.245	0.897	0.022
Chronic glomerulonephritis	0.672	0.495	0.913	0.011	0.676	0.436	1.049	0.081
Polycystic kidney disease	1.23	0.751	2.014	0.411	1.156	0.557	2.4	0.698
Other	0.746	0.488	1.139	0.175	0.816	0.454	1.47	0.499
Unknown	0.819	0.605	1.108	0.195	0.797	0.513	1.238	0.312
Spousal donor (vs. non-spousal)	1.687	1.349	2.11	<0.001	0.758	0.508	1.212	0.275
ABO incompatibility	1.572	1.18	2.094	0.002	1.499	1.001	2.245	0.05
Sensitization	1.437	0.967	2.138	0.073	1.413	0.857	2.327	0.175
HLA mismatch number (per 1 mismatch)	1.217	1.116	1.327	<0.001	1.21	1.079	1.356	0.001
Induction immunosuppression								
Anti-thymocyte globulin	Referent							
Basiliximab	0.837	0.498	1.406	0.5				
No induction	0.771	0.423	1.404	0.395				
Calcineurin inhibitors								
Cyclosporin	Referent							
Tacrolimus	0.987	0.76	1.281	0.922				
No use	2.151	0.988	4.682	0.054				
No steroid use	1.293	0.641	2.609	0.473				

HR = hazard ratio, CI = confidence interval, ESRD = end-stage renal disease, HLA = human leukocyte antigen.

### Allograft function between ABOi-KT and ABOc-KT from spousal donor

**Fig 5:**
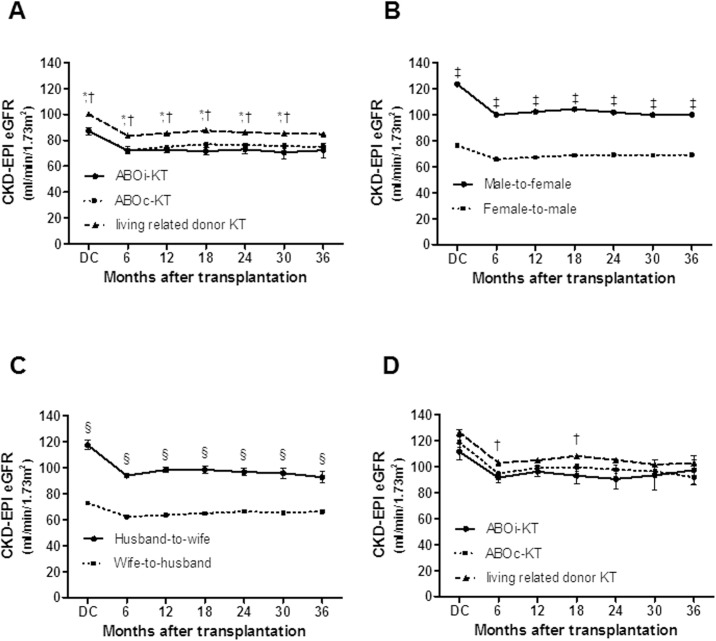
Comparison of allograft function. (A) Comparison of eGFR among spousal donor KT and living related donor KT. (B) Comparison of eGFR between male-to-female and female-to-male KT in total LD-KT. (C) Comparison of eGFR between husband-to-wife and wife-to-husband KT. (D) Comparison of eGFR of the male-to-female patients in the spousal donor KT and living related donor KT. ^*^, *P* < 0.05 for ABOi-KT from spousal donor vs. living related donor KT; ^†^, *P* < 0.05 for ABOc-KT from spousal donor vs. living related donor KT; ^‡^, *P* < 0.05 for male-to-female vs. female-to-male; ^§^, *P* < 0.05 for husband-to-wife vs. wife-to-husband. eGFR, estimated glomerular filtration rate; ABOi, ABO incompatible; KT, kidney transplantation; ABOc, ABO compatible;

We compared renal allograft function between spousal donor KT and living related donor KT and performed subgroup analysis according to the donor gender. Allograft function in spousal donor KT was lower than that of living related donor KT Fig 5A) but the male-to-female KT showed better allograft function than the female-to-male KT In living donor KT, (Fig 5B), and husband-to-wife KT showed better allograft function compared to the wife-to-husband KT in spousal donor KT, (Fig 5C). When we compare allograft function of male-to-female KT in spousal donor KT and living related donor KT, there was no significant difference between two groups (Fig 5D).

### Allograft and patient survival rate between ABOi-KT and ABOc-KT from spousal donor

**Fig 6:**
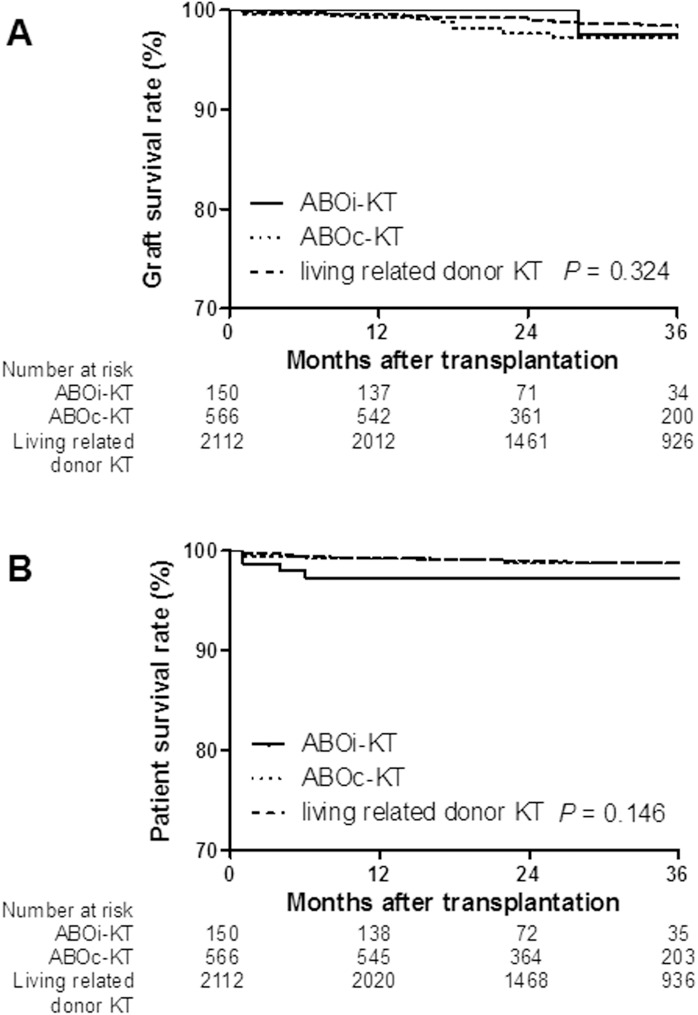
Comparisons of the graft and patient survival rates between ABOi- KT and ABOc-KT from spousal donor. (A) Graft survival rate. (B) Patient survival rate. The graft survival rates at 3-year post-transplantation showed no difference across the ABOi-KT, ABOc-KT from spousal donor and living related donor KT (96.3 vs. 96.7 vs. 97.9%, respectively). The patient survival rates at 3-year post-transplantation in ABOi-KT from spousal donor did not differ from ABOc-KT from spousal donor (97.3 vs. 98.4%) and comparable to living related donor KT (98.8%). ABOi, ABO incompatible; KT, kidney transplantation; ABOc, ABO compatible

Fifty five patients failed graft function during the study period. Three patients (2.0%) were the ABOi-KT from spousal donors, and 15 patients (2.7%) were the ABOc-KT from spousal donors. The overall graft survival for ABOi-KT from spousal donors at 3-year post-transplantation (96.3%) was not significantly different from the ABOc-KT (96.7%, *P* = 0.324; Fig 6A). BPAR was an independent risk factor for graft failure (*P* < 0.001), but spousal donor KT (*P* = 0.196) and ABOi-KT (*P* = 0.336) were not risk factors for graft failure (Table 3). In the matched analysis using a propensity score-matching algorithm, there was no difference in 3-year allograft survival between ABOi-KT and ABOc-KT from spousal donors (95.7 vs. 98.2%, *P* = 0.485).

**Table 3:** Univariate and multivariate cox regression analysis of risk factors for graft failure.

	Univariate				Multivariate			
	HR	95% CI	95% CI	*P*-value	HR	95% CI	95% CI	*P* -value
		Lower	Upper			Lower	Upper	
Recipient age (per 1 year-old)	0.991	0.972	1.011	0.393				
Donor age (per 1 year-old)	1.006	0.983	1.028	0.672				
Female recipient (vs. male)	0.868	0.517	1.457	0.592				
Female donor (vs. male)	0.85	0.514	1.404	0.526				
Retransplantation (vs. first)	0.296	0.041	2.136	0.227				
Duration of dialysis (per month)	1	0.993	1.007	0.985				
Causes of ESRD								
Diabetes mellitus	Referent							
Hypertension	0.448	0.167	1.2	0.11	0.55	0.203	1.491	0.24
Chronic glomerulonephritis	0.331	0.158	0.696	0.004	0.503	0.232	1.087	0.081
Polycystic kidney disease	0.876	0.298	2.574	0.809	1.381	0.452	4.213	0.571
Other	0.597	0.238	1.494	0.27	1.07	0.41	2.792	0.89
Unknown	0.542	0.279	1.054	0.071	0.838	0.418	1.68	0.619
Spousal donor (vs. non-spousal)	1.445	0.833	2.507	0.196				
ABO incompatibility	1.417	0.697	2.879	0.336				
Biopsy proven acute rejection	9.419	5.637	15.738	<0.001	8.19	4.841	13.854	<0.001
Sensitization	0.311	0.043	2.258	0.248				
HLA mismatch number (per 1 mismatch)	1.04	0.839	1.288	0.723				
Induction immunosuppression								
Anti-thymocyte globulin	Referent							
Basiliximab	2.408	0.333	17.411	0.387				
No induction	1.997	0.24	16.591	0.522				
Calcineurin inhibitors								
Cyclosporin	Referent							
Tacrolimus	0.789	0.41	1.521	0.48	0.877	0.453	1.698	0.697
No use	32.939	14.951	72.571	<0.001	9.611	3.127	29.536	<0.001
No steroid use	20.271	11.261	36.487	<0.001	7.436	2.697	20.504	< 0.001

HR = hazard ratio, CI = confidence interval, ESRD = end-stage renal disease, HLA = human leukocyte antigen.

The patient survival rate until 3 years post-transplantation showed no difference between spousal donor KT and living related donor KT, overall *P* = 0.146; Fig 6B). In the matched analysis between the ABOi-KT and ABOc-KT from spousal donors, there was no difference in at 3-year patient survival rate (96.9 vs. 98.8% *P* = 0.176).

The causes of death are shown in Table 4.

**Table 4:** Causes of death.

	ABOi-SD-KT	ABOc-SD-KT	LRD-KT
	(n = 150)	(n = 566)	(n = 2112)
Infection	2	3	11
Cardiovascular disease	0	1	3
Malignancy	0	1	2
Suicide	0	0	2
Other	1	3	3
Unknown	1	0	1
Total number of death	4	8	22

Overall *P* = 0.823

Abbreviations: ABOi = ABO incompatible, SD = spousal donor, KT = kidney transplantation, ABOc = ABO compatible, LRD = living related donor.

Infection was the most common cause of death in all three groups, followed by cardiovascular disease and malignancy. In the multivariate risk factor analysis, spousal donor was not a risk factor for death, but ABO incompatibility increased the risk for patient death 3.2-fold compared to the ABO compatible patients (*P* = 0.007). Other risk factors for patient death were older recipient age and no usage of calcineurin inhibitors or steroids (Table 5).

**Table 5:** Univariate and multivariate cox regression analysis of risk factors for patient death.

	Univariate				Multivariate			
	HR	95% CI	95% CI	*P* -value	HR	95% CI	95% CI	*P -*value
		Lower	Upper			Lower	Upper	
Recipient age (per 1 year-old)	1.075	1.04	1.111	<0.001	1.047	1.01	1.086	0.013
Donor age (per 1 year-old)	1.009	0.981	1.038	0.542				
Female recipient (vs. male)	1.048	0.551	1.996	0.886				
Female donor (vs. male)	0.968	0.512	1.829	0.919				
Retransplantation (vs. first)	2.7	1.054	6.916	0.038	0.946	0.22	4.059	0.94
Duration of dialysis (per month)	1.007	1.001	1.013	0.03	0.998	0.99	1.006	0.645
Causes of ESRD								
Diabetes mellitus	Referent							
Hypertension	1.431	0.593	3.455	0.425				
Chronic glomerulonephritis	0.42	0.169	1.044	0.062				
Polycystic kidney disease	0	0		0.982				
Other	0	0		0.973				
Unknown	0.58	0.247	1.367	0.213				
Spousal donor (vs. non-spousal)	1.542	0.778	3.057	0.215				
ABO incompatibility	3.574	1.801	7.092	<0.001	3.274	1.39	7.713	0.007
Biopsy proven acute rejection	1.884	0.861	4.121	0.113				
Sensitization	3.291	1.437	7.536	0.005	1.53	0.57	4.107	0.399
HLA mismatch number (per 1 mismatch)	1.238	0.965	1.587	0.093				
Induction immunosuppression								
Anti-thymocyte globulin	Referent							
Basiliximab	0.479	0.147	1.563	0.223				
No induction	0.218	0.036	1.305	0.095				
Calcineurin inhibitors								
Cyclosporin	Referent							
Tacrolimus	1.939	0.58	6.477	0.282	2.155	0.493	9.429	0.308
No use	107.046	30.351	377.545	<0.001	34.739	5.515	218.822	<0.001
No use of steroid	30.895	15.774	60.514	< 0.001	6.455	1.729	24.1	0.006

HR = hazard ratio, CI = confidence interval, ESRD = end-stage renal disease, HLA = human leukocyte antigen.

## Discussion

The results of our study clearly demonstrate that the introduction of ABOi-KT activated living donor KT, with the increase being more definite in spousal donor KT. Furthermore, the clinical outcomes of ABOi-KT from spousal donors were comparable to those of ABOc-KT. These findings suggest that ABOi-KT from spousal donors can be considered for end stage renal disease patients whose only potential donor is an ABO blood group mismatched spouse.

The most important finding in this study is that introduction of ABOi-KT increased overall living donor KT activity by 12.3%. When we started ABOi-KT, there were only 2 cases (0.3%) per year, but the number of ABOi-KT rapidly increased to 217 cases per year during 8 years, which accounted for 21.7% of the total living donor KT. Between living related and unrelated donor KT, The absolute number of ABOi-KT in living unrelated donor KT was less than living related donor KT (164 vs. 207) but its portion was two times higher than living related donor KT (17.8 vs. 9.8%). Our experience suggests that ABOi-KT is an important approach to overcome donor shortage by increasing both living related and unrelated donors KT, and its effect is more definite in living unrelated donors.

Our study focused on spousal donation after introduction of ABOi-KT because spouses have become important alternatives to living related donors in Korea. In our study, we confirmed that spouses were major portion of living unrelated donors (77.6%) and ABOi-KT increased its portion from 10% to 31.5% in living donor KT. Furthermore, the rate of increase of ABOi-KT was almost 10 times higher than that of living related donor KT (312 vs. 35%). This finding suggests that spousal donors are important source of living donor KT, and spousal donation will be more increased with introduction of ABOi-KT.

Most of previous reports about clinical outcomes of ABOi-KT or spousal donor KT were single center reports [3, 8, 11–14], and the specific analysis for ABOi-KT from spousal donors has not been widely studied yet. Using nationwide transplantation database, we found that the incidence of BPAR rate were not significantly different between the ABOi- KT and ABOc-KT from spousal donors. Similar results were observed in graft and patient survival rates. Moreover, spousal donor or ABO incompatibility was not an independent risk factor for the development of BPAR in the multivariate analysis. In addition, we observed that allograft function in spousal donor KT is affected by gender type rather than ABO incompatibility. [15] Taken together, results of our large database suggest that ABO incompatibility might not affect clinical outcomes in spousal donor KT.

Interestingly, most cases of mortality in ABOi-KT developed within 6 months post-transplantation, and the most common cause of death was infection. This finding was consistent with previous reports that strong pre-transplant desensitization increases infection-related mortality during the early post-transplantation period in ABOi-KT.[16–19] Recently, however, there is a trend to decrease desensitization intensity and maintenance immunosuppression dose; [8, 20–22 hence, we expect that the infection-related mortality might be decreased in the future.

This study has some limitations. First, the baseline or posttransplant anti-A/B antibody titers were not included. Second, we did not consider center effect. But, basic protocol including rituximab and plasmapheresis was similar. Third, recipients of our study group were too young (42.8 ± 12.6 years old) to represent general KT candidate. Forth, our data did not include information whether BPAR is due to antibody or T-cell mediated rejection. The last, we could not observe long-term clinical outcome due to short term follow-up (26 ± 10 months) in this cohort.

In conclusion, our study shows that the ABOi-KT contributed to the significant increase of living donor KT, and ABOi-KT from spousal donor is rapidly increasing with favorable clinical outcomes. These results suggest that ABOi-KT from spousal donors is a useful and acceptable alternative to overcome organ shortages.

## Supporting information

S1 File The law data from KONOS data base used in this study. (XLSX)

## Abbreviations

ABOc: ABO compatible
ABOi: ABO incompatible
BPAR: biopsy-proven acute rejection
CI: confidence interval
eGFR: estimated glomerular filtration rate
HLA: human leukocyte antigen
HR: hazard ratio
KT: kidney transplantation
